# Desempenho Individual e Combinado de Indicadores de Obesidade Geral e Central para Estimar Risco Coronariano em Participantes do ELSA-Brasil

**DOI:** 10.36660/abc.20200360

**Published:** 2021-07-21

**Authors:** Rogério Tosta de Almeida, Sheila Maria Alvim Matos, Estela M. L. Aquino

**Affiliations:** 1 Universidade Estadual de Feira de Santana Departamento de Saúde Feira de SantanaBA Brasil Universidade Estadual de Feira de Santana - Departamento de Saúde, Feira de Santana, BA – Brasil; 2 Universidade Federal da Bahia Instituto de Saúde Coletiva SalvadorBA Brasil Universidade Federal da Bahia - Instituto de Saúde Coletiva, Salvador, BA – Brasil

**Keywords:** Doenças Cardiovasculare, Antropometria, Obesidade, Fatores de Risco, Epidemiologia, Adiposidade, Índice de Massa Corporal, Circunferência da Cintura

## Abstract

**Fundamento:**

Indicadores antropométricos são utilizados na prática clínica e em estudos epidemiológicos para rastreamento de fatores de risco à saúde.

**Objetivo:**

Avaliar o poder discriminatório individual do Índice de Adiposidade Corporal (IAC), do Índice de Massa Corporal (IMC), da Circunferência da Cintura (CC) e da Razão Cintura-Quadril (RCQ) para identificar risco coronariano e investigar se a combinação de indicadores antropométricos de obesidade geral e central melhora a capacidade preditiva em adultos.

**Métodos:**

Avaliou-se 15 092 participantes (54,4% mulheres) com idades entre 35-74 anos na linha de base do ELSA-Brasil. Indivíduos em risco coronariano foram identificados pelo Escore de Risco de Framingham, e divididos em risco muito alto (RMA20%) e risco alto (RA10%). Medidas de acurácia diagnóstica e áreas sob curvas ROC (AUC) foram analisadas. Associações foram testadas por regressão de poisson com variância robusta, conforme sexo e idade. Foi adotada significância estatística de 5%.

**Resultados:**

A RCQ apresentou melhor poder discriminatório para RMA20% em todos os grupos, com maior capacidade preditiva nas mulheres (AUC: 0,802; IC_95%_: 0,748-0,856 *vs* 0,657; IC_95%_: 0,630-0,683 nas faixas etárias 35-59 anos e AUC: 0,668; IC_95%_: 0,621-0,715 *vs* 0,611; IC_95%_: 0,587-0,635 nas faixas etárias 60-74 anos). As combinações IAC+RCQ e IMC+RCQ apresentaram melhor poder preditivo em homens e mulheres, respectivamente. Combinações entre indicadores de obesidade geral e central estiveram mais fortemente associadas com RMA20% e RCA10% em todos os estratos.

**Conclusões:**

Indicadores combinados tiveram melhor capacidade preditiva do que um indicador isoladamente, sendo IAC+RCQ e IMC+RCQ melhores estimadores de risco coronariano em homens e mulheres, respectivamente. RCQ teve melhor desempenho individual.

## Introdução

A utilização de indicadores antropométricos de obesidade tem sido reconhecida como uma boa estratégia de rastreamento de fatores de risco à saúde, sendo muito empregada na prática clínica e em estudos epidemiológicos.^1^^-^^3^

O Índice de Massa Corporal (IMC), proposto por Lambert Adolphe Jaques Quetelet em 1832,^4^ é uma das estratégias mais divulgadas para medir indiretamente obesidade em populações. Apesar de ser bastante usado em estudos sobre doenças cardiovasculares,^3^^,^^5^ o IMC parece não ser capaz de descrever a ampla variação que ocorre na composição corporal de indivíduos.^3^

Nos últimos anos, novos indicadores têm sido propostos como medidas indiretas mais precisas de obesidade. Em 2011, Bergman et al.,^6^ sugeriram que o Índice de Adiposidade Corporal (IAC) era melhor indicador de gordura corporal do que o IMC. No entanto, a acurácia do IAC para medir adiposidade tem variado conforme a população estudada^7^^-^^9^ e os resultados do seu desempenho como estratégia de rastreamento de fatores de risco cardiometabólicos ainda são controversos.^10^^-^^16^ Os indicadores de distribuição da gordura corporal, como a Circunferência da Cintura (CC), a Razão Cintura-Quadril (RCQ), a Razão Cintura-Estatura (RCEst) e o Índice de Conicidade (índice C) têm demonstrado bons desempenhos como preditores de risco à saúde.^1^^,^^2^^,^^17^ Estudo recente com participantes do ELSA-Brasil demonstrou associação positiva entre CC e RCQ com espessura da camada íntima-média da parede da carótida.^17^ Ademais, o uso desses indicadores tem sido consolidado em estudos epidemiológicos, e não requer cálculos matemáticos complexos, como o índice C, por exemplo, o que justifica a opção em testá-los.

É controverso se os indicadores antropométricos de obesidade geral e os de obesidade central têm maior capacidade preditiva e utilidade para identificar fatores de risco cardiometabólicos em grandes populações.^18^^-^^22^ Além disso, enquanto alguns estudos indicam que o uso combinado de indicadores pode melhorar sua capacidade preditiva de desfechos adversos à saúde,^18^^,^^23^^-^^25^ outros concluem o contrário.^19^^,^^26^ Na população adulta brasileira, são escassos estudos sobre o desempenho combinado de indicadores de obesidade geral e central para rastrear desfechos adversos à saúde.

Assim, os objetivos deste estudo foram: 1) avaliar o poder discriminatório individual do IAC, do IMC, da CC e da RCQ para avaliar risco coronariano e, 2) investigar se a combinação de indicadores antropométricos de obesidade geral (IMC e IAC) com indicadores de obesidade central (CC e RCQ) melhora a capacidade preditiva de risco coronariano em uma grande amostra de adultos brasileiros.

## Métodos

### Fonte de dados e população do estudo

O Estudo Longitudinal de Saúde do Adulto (ELSA-Brasil) avaliou, na linha de base (2008-2010), 15 105 servidores de instituições de ensino e pesquisa de seis cidades brasileiras (Belo Horizonte, Porto Alegre, Rio de Janeiro, Salvador, São Paulo e Vitória), com idades entre 35-74 anos. Destes, nove participantes não tinham informações completas das variáveis analisadas e quatro foram excluídos por relatarem uso de prótese de silicone no quadril. Assim, o presente estudo avaliou 15 092 participantes. Os detalhes metodológicos sobre o ELSA-Brasil foram publicados por Aquino et al.^27^

### Dados demográficos, antropométricos e clínicos

Os dados demográficos, antropométricos e clínicos foram coletados por meio de entrevistas ou medidas, realizadas por uma equipe devidamente treinada e supervisionada por profissionais qualificados. Idade, sexo, tabagismo e história prévia de diabetes foram coletados por meio de questionários padronizados. As medidas antropométricas foram obtidas após jejum noturno de 8h-12h, com os participantes sem sapatos e de pé, vestidos com uniforme padronizado, fornecido pelo ELSA-Brasil. Um protocolo padrão, desenvolvido pelo estudo, com base nas recomendações da *International Society for the Advancement of Kinanthropometry* (ISAK),^28^ orientou as aferições.

O peso foi aferido com uma balança calibrada (Toledo 2096PP), aproximando-se para 0,1 kg mais próximo. A estatura foi medida com um estadiômetro (Seca-SE-216), e a CC e a circunferência do quadril (CQ) por uma trena antropométrica inelástica de 200cm (CESCORF), considerando o 0,1 cm mais próximo. A CC foi tomada no ponto médio entre a borda inferior do arco costal e a crista ilíaca, e a CQ na protusão máxima dos músculos glúteos (quadril). As medidas foram tomadas em triplicata e a média considerada para análise.

As amostras de sangue para dosagem de glicemia e de colesterol total e frações foram coletadas em tubos secos, após 12 horas de jejum noturno, nos próprios centros de investigação. A glicemia foi medida com método da hexoquinase (enzimático) e o colesterol com o método do colesterol oxidase (enzimático colorimétrico), ambos com equipamento calibrado e padronizado (Siemens ADVIA 1200). Para a garantia da qualidade e a padronização dos resultados, todas as amostras foram enviadas para processamento e análise em um laboratório central do ELSA-Brasil.

Foram feitas três medidas da pressão arterial com intervalo de um minuto após cinco minutos de repouso, utilizando-se aparelho automático de pressão arterial (OMRON - modelo HEM-705 CP), com os participantes sentados em ambiente silencioso com temperatura controlada (20ºC - 24ºC), em jejum e de bexiga vazia, seguindo protocolo padronizado desenvolvido pelo estudo. A média das duas últimas aferições foi considerada para análise.

### Indicadores antropométricos

Os indicadores antropométricos de obesidade geral testados foram: o IMC (Kg/m^2^) obtido dividindo-se o peso (Kg) pela estatura (m) ao quadrado e o IAC, calculado pela seguinte equação: IAC= [(circunferência do quadril em cm/estatura em metros)1,5 - 18.^6^ A obesidade central foi aferida por meio da medida de CC (cm) e da RCQ, calculada pela divisão da CC (cm) pela CQ (cm).

### Risco cardiovascular

O risco cardiovascular foi estimado pelo Escore de Risco de Framingham (ERF), e incluiu idade, Pressão Arterial Sistólica (PAS), Pressão Arterial Diastólica (PAD), colesterol total, lipoproteína de alta densidade (HDL)-colesterol, tabagismo e diabetes para seu cálculo.^29^

A presença de diabetes foi definida como: história prévia de diagnóstico médico da doença, glicemia de jejum ≥126 mg/dL, ou teste de tolerância oral à glicose 2h pós sobrecarga ≥200 mg/dL, ou hemoglobina glicada ≥6,5%.

Foram considerados fumantes aqueles que relataram o fumo atual de cigarros na entrevista.

O risco cardiovascular aumentado foi classificado em: risco muito aumentado (RMA), se o indivíduo apresentasse um risco maior que 20% de desenvolvimento de Doença Arterial Coronariana (DAC) em 10 anos (RMA20%); ou risco aumentado (RA) se o indivíduo apresentasse um risco entre 10 e 20% de desenvolvimento de DAC em 10 anos (RA10%).

### Análise estatística

As medidas de tendência central e dispersão foram utilizadas para análises iniciais da distribuição dos participantes. A normalidade dos dados foi avaliada descritivamente, por análise gráfica (histograma e P-Plot) e teste de Shapiro Wilk. Analisou-se a homogeneidade da variância pelo teste F. Variáveis contínuas com distribuição normal foram expressas em média e desvio padrão e testadas com o teste *t student* não pareado. Aquelas com distribuição não normal foram expressas em mediana e intervalo interquartil e avaliadas com o teste de *Mann-Whitney*. Variáveis categóricas foram testadas com *chi-quadrado de Pearson* e expressas em frequências absolutas e relativas.

Na sequência, foram determinados os pontos de corte dos indicadores (IMC, IAC, RCQ e CC) para discriminar 20% a mais de risco coronariano em 10 anos (RMA20%), por meio das curvas ROC (*Receiver Operating Characteristic*), conforme sexo e faixa etária. Utilizou-se o maior valor do índice de *Youden* (sensibilidade + especificidade - 1) como critério para escolha do melhor ponto. As áreas sob as curvas ROC (AUCs), com os respectivos Intervalos de Confiança de 95% (IC95%), foram usadas para avaliar a capacidade preditiva individual e combinada dos indicadores. As AUCs foram comparadas pelo teste não paramétrico de Delong et al.,^30^ sendo checadas as diferenças dos indicadores individuais e combinados entre os sexos e entre as faixas etárias. Ainda, foram comparadas as AUC da melhor combinação de indicadores com as AUC do melhor indicador individual em cada grupo.

A capacidade discriminatória foi testada individualmente e por combinações entre um indicador de obesidade geral com outro de obesidade central. Primeiramente, quatro combinações foram analisadas (IAC+CC, IMC+CC, IAC+RCQ e IMC+RCQ), pontuadas e agrupadas da seguinte forma: 0 = sem valores aumentados de ambos indicadores; 1 = valor aumentado apenas do indicador de obesidade geral; 2 = valor aumentado apenas do indicador de obesidade central; e 3 = valor aumentado de ambos. Por exemplo, na combinação IAC+CC, os grupos foram: “0 = IAC_0_+CC_0_”; “1 = IAC_1_+CC_0_”; “2 = IAC_0_+CC_1_”; e “3 = IAC_1_+CC_1_”.

Em seguida, para avaliar a associação ajustada entre os indicadores de obesidade geral, de obesidade central e a combinação de ambos, novo reagrupamento em quatro categorias foi realizado, de nenhum indicador aumentado a pelo menos um indicador central e um geral juntos (isto é, 00, 01, 10, 11). Para isso, variáveis *dummies* foram criadas, sendo: 0 = participantes sem valores aumentados de nenhum dos indicadores analisados; 1 = aqueles que apresentaram, pelo menos, um indicador geral aumentado e nenhum central aumentado; 2 = participantes que apresentaram, pelo menos, um indicador central aumentado e nenhum geral; e 3 = as outras possíveis combinações entre, pelo menos, um indicador central e pelo menos um indicador geral aumentados.

As associações foram testadas por modelos de regressão de Poisson com variância robusta, observando-se as Razões de Prevalências (RP) e os IC95% entre as combinações dos indicadores e o RMA20%. Em função da frequência do RMA20% ser muito baixa nas mulheres mais jovens (0,8%), bem como, a quantidade de participantes em risco ser pequena em algumas combinações, realizou-se análise adicional para confirmação ou não das associações individuais e combinadas dos indicadores antropométricos com risco coronariano, sendo o desfecho o RCA10%.

O ajuste dos modelos foi verificada pela análise dos resíduos de Pearson.

O nível de significância adotado foi de 5% (p<0,05). As análises estatísticas foram realizadas utilizando o *software* “STATA”, versão 12.0.

## Resultados

Foram investigados 6881 homens (28,5% com idade entre 60-74 anos) e 8211 mulheres (26,7% com idade entre 60-74 anos) da linha de base do ELSA-Brasil. As médias de colesterol total, PAS e PAD e as medianas de idade e HDL-colesterol foram mais altas nos participantes com idade entre 60-74 anos comparados com os mais jovens em ambos os sexos, com exceção da PAD e do colesterol total nos homens.

As prevalências de RMA20% nos homens foram de 6,3% e 40,6% e nas mulheres de 0,8% e 6,1%, nas faixas etárias 35-59 e 60-74 anos, respectivamente. Os homens apresentaram maiores frequências de obesidade central (CC_1_ e RCQ_1_) do que as mulheres, em ambas as faixas etárias, sendo mais frequentes na faixa etária de 60-74 anos em ambos os sexos. As diferenças entre os percentuais de participantes com obesidade geral variaram conforme o indicador observado (tabela 1).

**Tabela 1:** Características dos participantes, conforme sexo e faixa etária – ELSA-Brasil (2008-2010)

	Homens		Mulheres	
Variável	35-59 anos (n= 5.354)	60-74 anos (n= 1.527)	35-59 anos (n= 6.478)	60-74 anos (n= 1.733)
Idade, mediana (IIQ)	48 (44-54)*	65 (62-69)*†	49 (44-54)	64 (61-68)†
Colesterol Total, média (DP)	214,1 (44,6)	207,1 (42,4)*†	214,5 (39,8)	224,2 (45,4)†
HDL-Colesterol, mediana (IIQ)	48 (42-56)*	50 (43-59)*†	59 (51-70)	62 (52-73)†
Pressão Arterial Sistólica, média (DP)	124,0 (15,7)*	131,1 (19,2)*†	115,1 (15,3)	127,4 (19,1)†
Pressão Arterial Diastólica, média (DP)	79,2 (10,8)*	77,9 (10,9)*†	73,8 (10,2)	74,8 (10,2)†
RCA10%, n (%)	1.711 (32,0)*	1.285 (84,2)*†	542 (8,4)	613 (35,4%)†
RMA20%, n (%)	340 (6,3)*	620 (40,6)*†	52 (0,8)	106 (6,1)†
Diabetes, n (%)	1.301 (19,3)*	569 (37,3)*†	882 (13,6)	484 (27,9)†
Fumantes, n (%)	813 (15,2)*	171 (11,2)*†	855 (13,2)	138 (7,9)†
IAC_1,_ n (%)	1.521 (28,4)*	524 (34,3)†	2.468 (38,1)	585 (33,8)†
IMC_1_, n (%)	2.425 (45,3)*	542 (35,5)†	2.269 (35,0)	583 (33,6)
CC_1,_ n (%)	2.491 (46,5)*	871 (57,0)*†	2.346 (36,2)	682 (39,3)†
RCQ_1,_ n (%)	1.761 (32,9)*	892 (58,4)*†	1.351 (20,9)	598 (34,5)†

HDL: lipoproteína de alta densidade; RCA10%: Risco Coronariano Aumentado; RMA20%: Risco Coronariano Muito Aumentado; IAC: Índice de Adiposidade Corporal; IMC: Índice de Massa Corporal; CC: Circunferência da Cintura; RCQ: Razão Cintura-Quadril; DP: Desvio Padrão; IIQ: Intervalo Interquartil; n (%): número de observações (frequência)

* p< 0,05 comparado com mulheres da mesma faixa etária;

† p< 0,05 comparado com os mais jovens do mesmo sexo

Os valores dos melhores pontos de corte para cada indicador estimar RMA20% e estabelecer os agrupamentos estão listados na Tabela 2, onde “0” significa “normal” e “1” “aumentado”.

**Tabela 2:** Pontos de corte de indicadores antropométricos e classificações correspondentes – ELSA-Brasil (2008-2010)

Indicador	Ponto de corte	Categoria	Classe*
IAC	28 (homens) 34 (mulheres 35-59 anos) 36 (mulheres 60-74 anos)	IAC < 28 (homens) ou IAC < 34 (mulheres 35-59 anos) ou IAC < 36 (mulheres 60-74 anos)	IAC_0_
		IAC ≥ 28 (homens) ou IAC ≥ 34 (mulheres 35-59 anos) ou IAC ≥ 36 (mulheres 60-74 anos)	IAC_1_
IMC	27 (homens 35-59 anos) 28 (homens 60-74 anos) 28 (mulheres 35-59 anos) 29 (mulheres 60-74 anos)	IMC < 27 (homens 35-59 anos) ou IMC < 28 (homens 60-74 anos) ou IMC < 34 (mulheres 35-59 anos) ou IMC < 36 (mulheres 59-74 anos)	IMC_0_
		IMC ≥ 27 (homens 35-59 anos) ou IMC ≥ 28 (homens 60-74 anos) ou IMC ≥ 34 (mulheres 35-59 anos) ou IMC ≥ 36 (mulheres 60-74 anos)	IMC_1_
CC	95 (homens) 90 (mulheres 35-59 anos) 93 (mulheres 60-74 anos)	CC < 95 (homens) ou CC < 90 (mulheres 35-59 anos) ou CC < 93 (mulheres 60-74 anos)	CC_0_
		CC ≥ 95 (homens) ou CC ≥ 90 (mulheres 35-59 anos) ou CC ≥ 93 (mulheres 60-74 anos)	CC_1_
RCQ	0,97 (homens) 0,90 (mulheres 35-59 anos) 0,91 (mulheres 60-74 anos)	CC < 0,97 (homens) ou CC < 0,90 (mulheres 35-59 anos) ou CC < 0,91 (mulheres 60-74 anos)	RCQ_0_
		CC ≥ 0,97 (homens) ou CC ≥ 0,90 (mulheres 35-59 anos) ou CC ≥ 0,91 (mulheres 60-74 anos)	RCQ_1_

IAC: Índice de Adiposidade Corporal; IMC: Índice de Massa Corporal; CC: Circunferência da Cintura; RCQ: Relação Cintura-Quadril.

* “0 = normal” e “1 = aumentado”.

A RCQ foi, individualmente, o indicador antropométrico que melhor discriminou RMA20% em todos os grupos. A AUC da combinação IAC+RCQ foi maior nos homens e da combinação IMC+RCQ foi maior nas mulheres, de ambas as faixas etárias. O desempenho de todos indicadores individuais ou combinados foi melhor nas mulheres do que nos homens, com exceção do IAC_1_, que não apresentou diferença estatística. De forma similar, na comparação entre participantes de faixas etárias diferentes, somente o IAC_1_ em ambos os sexos e o IMC_1_ nos homens não apresentaram diferença estatística.

As análises combinadas apresentaram valores de sensibilidade maiores do que cada indicador individualmente, com exceção das combinações IAC+CC e IMC+CC nos homens mais idosos, que mostraram sensibilidade um pouco menor que a RCQ. Os valores de sensibilidade de todas as combinações ficaram acima de 70%, atingindo valores acima de 90% nas mulheres na faixa etária de 35 -59 anos (Tabela 3).

**Tabela 3:** Medidas de acurácia diagnóstica de indicadores antropométricos de obesidade individuais e combinados para estimar 20% de risco coronariano em 10 anos, conforme sexo e faixa etária – ELSA-Brasil (2008-2010)

	Homens 35-59 anos (n=5.354)			Homens 60-74 anos (n=1.527)					
Indicadores	SEN (%)	ESP (%)	AUC (IC 95%)	SEN (%)		ESP (%)		AUC (IC 95%)	p
**Individuais**									
*IAC*_*1*_	43,2	72,6	0,578 (0,551 – 0,605)	39,8		69,5		0,547 (0,522 – 0,571)	0,080
*IMC*_*1*_	62,9	55,9	0,594 (0,568 – 0,621)	43,2		69,8		0,565 (0,541 – 0,590)	0,116
*CC*_*1*_	64,9	54,7	0,598 (0,572 – 0,624)	64,2		47,9		0,560 (0,535 – 0,585)	0,041
*RCQ*_*1*_	62,2	69,1	**0,657 (0,630 – 0,683)** a b c	71,6		50,6		**0,611 (0,587 – 0,635)** a b c	0,013
**Combinações**									
*IAC + CC*	70,9	49,3	0,618 (0,589 – 0,647)	70,3	41,7		0,574 (0,549 – 0,601)		0,029
*IAC + RCQ*	75,3	55,7	**0,680 (0,652 – 0,707)**^1^,^3^#	80,3	40,0		**0,624 (0,597 – 0,650)**^1^,^3^		0,004
*IMC + CC*	71,2	48,3	0,610 (0,583 – 0,638)	64,6	46,6		0,578 (0,550 – 0,605)		0,010
*IMC + RCQ*	77,9	48,4	0,672 (0,645 – 0,700)^2^,^4^	76,0	44,4		0,623 (0,596 – 0,650)^2^,^4^		0,013
	**Mulheres 35-59 anos (n=6.478)**			**Mulheres 60-74 anos (n=1.733)**					
**Indicadores**	**SEN (%)**	**ESP (%)**	**AUC (IC 95%)**	**SEN (%)**	**ESP (%)**		**AUC (IC 95%)**		**p**
**Individuais**									
*IAC*_*1*_	63,5	62,1	0,628 (0,562 – 0,694)	50,9	67,4		0,592 (0,542 – 0,641)		0,389
*IMC*_*1*_	78,9	65,3	0,721 (0,665 – 0,777)^e^*	58,5	68,0		0,632 (0,584 – 0,681)*		0,020
*CC*_*1*_	92,3	64,2	0,783 (0,746 – 0,820)^d^^f^*	68,9	62,6		0,657 (0,611 – 0,703)^d^*		<0,001
*RCQ*_*1*_	80,8	79,6	**0,802 (0,748 – 0,856)** a b *	66,0	67,6		**0,668 (0,621 – 0,715)** a *		<0,001
**Combinações**									
*IAC + CC*	94,2	51,6	0,777 (0,734 – 0,820)*	75,5	52,6		0,667 (0,618 – 0,717)*		0,001
*IAC + RCQ*	98,1	53,2	0,846 (0,810 – 0,882)^1^,^3^*	85,8	48,1		0,709 (0,663 – 0,754)*		<0,001
*IMC + CC*	92,3	59,0	0,786 (0,743 – 0,829)*	70,8	57,9		0,668 (0,618 – 0,717)*		<0,001
*IMC + RCQ*	96,2	59,4	**0,850 (0,811 – 0,889)**^2^,^4^*#	82,1	52,1		**0,710 (0,663 – 0,757)** 4 * #		<0,001

IAC: Índice de Adiposidade Corporal; IMC: Índice de Massa Corporal; CC: Circunferência da Cintura; RCQ: Relação Cintura-Quadril; SEN: Sensibilidade; ESP: Especificidade; AUC: Área sob as curvas ROC; IC_95%_: Intervalo de Confiança 95%; p: valor de p na comparação entre faixas etárias do mesmo sexo. **Negrito**: Maiores valores de AUC em cada agrupamento.

p≤0,05 entre indicadores individuais por sexo

a RCQ > IAC;

b RCQ > IMC;

c RCQ > CC;

d CC > IAC;

e IMC > IAC;

f CC > IMC; entre combinações por sexo

1 IAC+RCQ > IAC+CC;

2 IMC+RCQ > IAC+CC;

3 IAC+RCQ > IMC+CC;

4 IMC+RCQ > IMC+CC;

* com homens da mesma faixa etária;

# melhor combinação com melhor individual.

**Figura 1:**
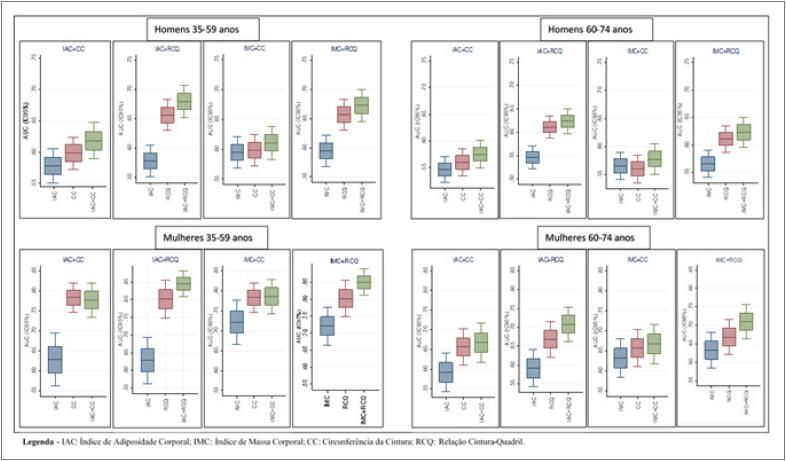
Representação gráfica das áreas sob as curvas ROC de indicadores antropométricos individuals e combinados para identificar risco coronariano muito aumentado, conforme sexo e faixa etária. ELSA-Brasil 2008-2010.

A capacidade preditiva de dois indicadores juntos foi melhor do que quando avaliado um indicador isoladamente. Por exemplo, as AUCs do IMC_1_ e da RCQ_1_ nas mulheres da faixa etária 60-74 anos foram, respectivamente, 0,632 e 0,668, mas, a AUC da combinação IMC+RCQ foi de 0,710, tendo um aumento de, pelo menos, 0,042 (0,042/0,668 = 6,3%). Os ganhos com as melhores combinações variaram entre 2,1% e 6,3% (Figura 1).

As combinações entre um indicador de obesidade geral e um de obesidade central (IAC_1_+CC_1_, IAC_1_+RCQ_1_, IMC_1_+CC_1_ e IMC_1_+RCQ_1_) estiveram associadas positivamente com RMA20% em homens e mulheres de ambas as faixas etárias, independente da presença de apenas um indicador, quer seja ele, indicador geral ou central. As razões de prevalências dessas combinações também foram maiores do que as demais em todos os grupos (Tabela 4).

**Tabela 4:** Razões de prevalências ajustadas de combinações de indicadores antropométricos de obesidade para estimar risco coronariano muito aumentado, conforme sexo e faixa etária no ELSA-Brasil (2008-2010)

	Homens				Mulheres			
	35-59 anos (n=5354)		60-74 anos (n=1527)		35-59 anos (n=6478)		60-74 anos (n=1733)	
Combinações de indicadores	n (risco)	RP (IC_95%_)	n (risco)	RP (IC_95%_)	n (risco)	RP (IC_95%_)	n (risco)	RP (IC_95%_)
IAC_0_ + CC_0_	2.573 (99)	1,0	562 (184)	1,0	3.321 (3)	1,0	881 (26)	1,0
IAC_1_ + CC_0_	290 (20)	1,79 (1,13 – 2,85)	94 (38)	1,23 (0,94 – 1,62)	811 (1)	1,36 (0,14 – 13,11)	170 (7)	1,40 (0,62 – 3,16)
IAC_0_ + CC_1_	1.260 (94)	1,94 (1,47 – 2,55)	441 (189)	1,31 (1,12 – 1,54)	689 (16)	25,70 (7,51 – 87,99)	267 (26)	3,30 (1,95 – 5,58)
IAC_1_ + CC_1_	1.231 (126)	2,66 (2,06 – 3,43)	430 (209)	1,48 (1,27 – 1,73)	1.657 (32)	21,38 (6,55 – 69,72)	415 (47)	3,84 (2,41 – 6,11)
								
IAC_0_ + RCQ_0_	2.876 (84)	1,0	485 (122)	1,0	3.420 (1)	1,0	798 (15)	1,0
IAC_1_ + RCQ_0_	717 (44)	2,10 (1,47 – 3,00)	150 (54)	1,43 (1,10 – 1,86)	1.707 (9)	18,03 (2,29 – 142,22)	337 (21)	3,32 (1,73 – 6,35)
IAC_0_ + RCQ_1_	957 (109)	3,90 (2,96 – 5,13)	518 (251)	1,93 (1,61 – 2,30)	590 (18)	104,33 (13,95 – 780,18)	350 (37)	5,62 (3,13 – 10,11)
IAC_1_ + RCQ_1_	804 (102)	4,34 (3,29 – 5,74)	374 (193)	2,05 (1,71 – 2,46)	761 (24)	107,85 (14,62 – 796,13)	248 (33)	7,08 (3,91 – 12,82)
								
IMC_0_ + CC_0_	2.521(98)	1,0	642 (219)	1,0	3.795 (4)	1,0	973 (31)	1,0
IMC_1_ + CC_0_	342 (21)	1,58 (1,00 – 2,50)	14 (3)	0,63 (0,23 – 1,72)	337 (0)	----	78 (2)	0,80 (0,20 – 3,30)
IMC_0_ + CC_1_	408 (28)	1,76 (1,17 – 2,65)	343 (133)	1,14 (0,96 – 1,35)	414 (7)	16,04 (4,71 – 54,61)	177 (13)	2,31 (1,23 – 4,32)
IMC_1_ + CC_1_	2.083 (192)	2,37 (1,87 – 3,00)	528 (265)	1,47 (1,28 – 1,69)	1.932 (41)	20,14 (7,22 – 56,19)	505 (60)	3,73 (2,45 - 5,68)
								
IMC_0_ + RCQ_0_	2.500 (75)	1,0	552 (149)	1,0	3.820 (2)	1,0	866 (19)	1,0
IMC_1_ + RCQ_0_	1.093 (53)	1,62 (1,15 – 2,28)	83 (27)	1,21 (0,86 – 1,69)	1.307 (8)	11,69 (2,48 – 54,99)	269 (17)	2,88 (1,52 – 5,46)
IMC_0_ + RCQ_1_	429 (51)	3,96 (2,82 – 5,57)	433 (203)	1,74 (1,46 – 2,06)	389 (9)	44,19 (9,58 – 203,82)	284 (25)	4,01 (2,24 – 7,18)
IMC_1_ + RCQ_1_	1.332 (160)	4,00 (3,07 – 5,22)	459 (241)	1,95 (1,65 – 2,19)	962 (33)	65,52 (15,75 – 272,60)	314 (45)	6,53 (3,88 – 10,99)

IAC: Índice de Adiposidade Corporal; IMC: Índice de Massa Corporal; CC: Circunferência da Cintura; RCQ: Relação Cintura-Quadril; RP: Razão de Prevalência; IC_95%_: Intervalo de Confiança 95%; n: total de participantes no estrato; risco: quantidade de participantes com risco coronariano muito aumentado no estrato.

Embora a prevalência de mulheres com RMA20% tenha sido baixa na idade entre 35-59 anos (menos de 1%), o número de mulheres tomadas como referências (sem risco) foi muito pequeno, o que explica os enormes intervalos de confiança apresentados. Além disso, em alguns estratos, a quantidade de participantes em risco foi pequena, como por exemplo, na combinação IMC_1_+CC_0_ nos homens e mulheres mais idosos, dificultando a avaliação nestes grupos. Desta forma, avaliamos ainda as associações das combinações de indicadores antropométricos com RA10%, que confirmaram os resultados que pessoas com medidas aumentadas de um indicador de obesidade geral (IAC ou IMC) e outro de obesidade central (CC ou RCQ) têm maior probabilidade de desenvolvimento de DAC em 10 anos (Tabela 5).

**Tabela 5:** Razões de prevalências ajustadas de combinações de indicadores antropométricos de obesidade para estimar risco coronariano aumentado, conforme sexo e faixa etária. ELSA-Brasil (2008-2010)

	Homens				Mulheres			
	35-59 anos (n=5.354)		60-74 anos (n=1.527)		35-59 anos (n=6.478)		60-74 anos (n=1.733)	
Combinações de indicadores	n (risco)	RP (IC_95%_)	n (risco)	RP (IC_95%_)	n (risco)	RP (IC_95%_)	n (risco)	RP (IC_95%_)
**IAC_0_ + CC_0_**	2.573 (559)	1,0	562 (430)	1,0	3.321 (137)	1,0	881 (213)	1,0
**IAC_1_ + CC_0_**	290 (94)	1,49 (1,24 – 1,79)	94 (77)	1,07 (0,96 – 1,19)	811 (35)	1,05 (0,73 – 1,50)	170 (49)	1,19 (0,92 – 1,55)
**IAC_0_ + CC_1_**	1.260 (485)	1,77 (1,60 – 1,96)	441 (386)	1,14 (1,08 – 1,21)	689 (106)	3,73 (2,93 – 4,74)	267 (132)	2,04 (1,73 – 2,42)
**IAC_1_ + CC_1_**	1.231 (572)	2,14 (1,94 – 2,35)	430 (209)	1,19 (1,13 – 1,26)	1.657 (264)	3,86 (3,17 – 4,71)	415 (219)	2,18 (1,88 – 2,53)
								
**IAC_0_ + RCQ_0_**	2.876 (607)	1,0	485 (345)	1,0	3.420 (125)	1,0	798 (161)	1,0
**IAC_1_ + RCQ_0_**	717(239)	1,58 (1,39 – 1,79)	150 (127)	1,19 (1,09 – 1,30)	1.707 (117)	1,88 (1,47 – 2,40)	337 (122)	1,79 (1,47 – 2,19)
**IAC_0_ + RCQ_1_**	957 (437)	2,16 (1,96 – 2,39)	518 (471)	1,28 (1,20 – 1,36)	590 (118)	5,47 (4,32 – 6,93)	350 (184)	2,61 (2,20 – 3,09)
**IAC_1_ + RCQ_1_**	804 (427)	2,52 (2,29 – 2,77)	374 (342)	1,29 (1,20 – 1,37)	761 (182)	6,54 (5,28 – 8,10)	248 (146)	2,92 (2,45 – 3,47)
								
**IMC_0_ + CC_0_**	2.521 (562)	1,0	642 (498)	1,0	3.795 (146)	1,0	973 (233)	1,0
**IMC_1_ + CC_0_**	342 (91)	1,19 (0,99 – 1,44)	14 (9)	0,83 (0,56 – 1,23)	337 (26)	2,00 (1,34 – 3,00)	78 (29)	1,55 (1,14 – 2,12)
**IMC_0_ + CC_1_**	408 (154)	1,69 (1,47 – 1,96)	343 (299)	1,12 (1,06 – 1,19)	414 (57)	3,58 (2,68 – 4,78)	177 (85)	2,01 (1,66 – 2,42)
**IMC_1_ + CC_1_**	2.083 (903)	1,94 (1,78 – 2,12)	528 (479)	1,17 (1,11 – 1,23)	1.932 (313)	4,21 (3,49 – 5,09)	505 (266)	2,20 (1,91 – 2,53)
								
**IMC_0_ + RCQ_0_**	2.500 (512)	1,0	552 (401)	1,0	3.820 (131)	1,0	866 (172)	1,0
**IMC_1_ + RCQ_0_**	1.093 (334)	1,49 (1,33 – 1,68)	83 (71)	1,18 (1,06 – 1,30)	1.307 (111)	2,48 (1,94 – 3,16)	269 (111)	2,08 (1,71 – 2,53)
**IMC_0_ + RCQ_1_**	429 (204)	2,32 (2,05 – 2,63)	433 (396)	1,26 (1,19 – 1,34)	389 (72)	5,40 (4,13 – 7,06)	284 (146)	2,59 (2,17 – 3,08)
**IMC_1_ + RCQ_1_**	1.332 (660)	2,42 (2,20 – 2,66)	459 (417)	1,25 (1,18 – 1,33)	962 (228)	6,91 (5,64 – 8,47)	314 (184)	2,95 (2,51 – 3,47)

* IAC: Índice de Adiposidade Corporal; IMC: Índice de Massa Corporal; CC: Circunferência da Cintura; RCQ: Relação Cintura-Quadril; RP: Razão de Prevalência; IC_95%_: Intervalo de Confiança 95%; n: total de participantes no estrato; risco: quantidade de participantes com risco coronariano aumentado no estrato.

A presença de obesidade central (CC_1_ e/ou RCQ_1_), na ausência de obesidade geral (IMC_1_ e/ou IAC_1_) esteve mais fortemente associada com risco coronariano aumentado e muito aumentado do que a combinação contrária. A magnitude do efeito da presença de obesidade geral e central juntas foi maior em todos os estratos, com exceção na associação com RMA20% nos homens mais jovens (Tabela 6).

**Tabela 6:** Razões de prevalências de indicadores antropométricos de obesidade geral e central para estimar risco coronariano muito aumentado e aumentado em 10 anos, conforme sexo e faixa etária. ELSA-Brasil (2008-2010)

	Risco coronariano muito aumentado (RMA20%)							
	Homens				Mulheres			
	35-59 anos (n=5354)		60-74 anos (n=1527)		35-59 anos (n=6478)		60-74 anos (n=1733)	
Combinações de indicadores	n (risco)	RP (IC_95%_)	n (risco)	RP (IC_95%_)	n (risco)	RP (IC_95%_)	n (risco)	RP (IC_95%_)
Sem risco	2.201 (63)	1,0	421 (111)	1,0	3.079 (1)	1,0	721 (13)	1,0
Apenas Geral	438 (23)	1,83 (1,15 – 2,92)	81 (28)	1,31 (0,93 – 1,84)	850 (1)	3,62 (0,23 – 57,79)	168 (7)	2,31 (0,97 – 5,70)
Apenas Central	585 (55)	3,28 (2,31 – 4,66)	440 (199)	1,72 (1,42 – 2,07)	509 (8)	48,39 (6,06 – 386,16)	303 (25)	4,58 (2,37 – 8,83)
Central + Geral	2.130 (198)	3,25 (2,46 – 4,28)	585 (282)	1,83 (1,53 – 2,19)	2.039 (42)	63,42 (8,73 – 460,51)	541 (61)	6,25 (3,47 – 11,26)
								
**Risco coronariano aumentado (RCA10%)**								
Sem risco	2.201 (425)	1,0	421 (300)	1,0	3.079 (103)	1,0	721 (135)	1,0
Apenas Geral	438 (118)	1,40 (1,17 – 1,66)	81 (63)	1,09 (0,96 – 1,24)	851 (30)	1,05 (0,71 – 1,57)	168 (48)	1,53 (1,15 – 2,03)
Apenas Central	585 (242)	2,14 (1,88 – 2,47)	440 (393)	1,25 (1,17 – 1,34)	509 (80)	4,70 (3,56 – 6,20)	303 (144)	2,54 (2,09 – 3,08)
Central + Geral	2.130 (925)	2,25 (2,04 – 2,48)	585 (529)	1,27 (1,19 – 1,36)	2.039 (329)	4,82 (3,89 – 5,98)	541 (286)	2,82 (2,38 – 3,35)

RP: Razão de Prevalência; IC_95%_: Intervalo de Confiança 95%; n: total de participantes no estrato; risco: quantidade de participantes com risco coronariano futuro no estrato.

## Discussão

O IAC_1_ demonstrou capacidade preditiva apenas razoável em todos os grupos investigados, com AUC variando entre 0,547 (IC_95%_: 0,522-0,571) nos homens na faixa etária 60-74 anos, e 0,628 (IC_95%_: 0,562-0,694) nas mulheres mais jovens. Esse resultado foi semelhante ao IMC_1_ que, apesar dos valores de AUCs ligeiramente maiores que as AUCs do IAC_1_ apenas no grupo das mulheres mais jovens, essa diferença foi estatisticamente significante (p<0,05). O indicador antropométrico com melhor desempenho individual em todos os grupos foi a RCQ.

As curvas ROC são frequentemente utilizadas para avaliar o desempenho de um teste diagnóstico, e, provavelmente, constituem a análise estatística mais usada para medir a capacidade preditiva de indicadores antropométricos. Todavia, muitos estudos não realizam a comparação estatística entre os indicadores ou grupos analisados, restringindo-se à observação da maior AUC, dificultando assim, melhor avaliação do seu desempenho. Contudo, Xiao et al.,^12^ comparando as AUCs, assinalaram que a capacidade discriminatória de diabetes mellitus tipo II (DM2), do IAC e do IMC não foram diferentes estatisticamente entre mulheres e homens chineses, e que os indicadores de obesidade central – CC, RCEst e RCQ – foram melhores preditores de DM2 nas mulheres que nos homens.^12^ Similarmente, os resultados da presente análise demonstraram que IMC e IAC tiveram desempenhos parecidos em ambos os sexos e CC e RCQ estimaram melhor risco coronariano em mulheres.

Leal Neto et al.,^14^ não encontraram diferenças estatísticas entre as AUCs do IAC e do IMC (p=0,885) para discriminar hipertensão em idosos brasileiros de ambos os sexos, apesar das AUCs ligeiramente maiores do IAC que do IMC. Resultado similar, na comparação entre IAC e IMC, foi encontrado em mulheres colombianas para identificar níveis elevados de pressão arterial e glicemia e síndrome metabólica,^10^ e para incidência de hipertensão em trabalhadores participantes do *Olivetti Heart* Study.^31^ Entretanto, o IMC foi melhor do que o IAC para indicar síndrome metabólica em mulheres coreanas.^32^

Por outro lado, Alvim et al.,^13^ evidenciaram o IAC como melhor preditor de DM2 em índias e índios brasileiros do que a CC e o IMC. No entanto, em amostra da população geral, residente em uma capital brasileira, os desempenhos do IAC, do IMC e da CC foram similares entre os homens e a CC foi melhor preditor de DM2 do que IAC e IMC nas mulheres.

Em estudo longitudinal com 2981 iranianos, IAC e IMC tiveram desempenhos similares como preditores de DM2 em homens, sendo que o IAC não foi bom preditor em mulheres (AUC=0,527; IC_95%_: 0,484-0,569). O poder preditivo da RCQ foi razoável em ambos os sexos, sendo pior do que a CC e a RCEst nos homens e semelhante aos demais indicadores nas mulheres.^33^

O risco coronariano analisado de forma global ainda é um desfecho pouco investigado, dificultando a comparação dos resultados desse estudo com outros achados. Todavia, Felix-Redondo et al.,^22^ estudando 28 743 espanhóis, encontraram associação positiva do IMC, CC e RCQ com eventos coronarianos futuros, calculado pelo escore REGICOR, adaptado do ERF e validado para população espanhola. Almeida et al.,^15^ identificaram associação positiva do IAC com ERF em 14 673 participantes do ELSA-Brasil.

Wang et al.,^34^ analisaram o desempenho individual de oito indicadores antropométricos para estimar risco coronariano – avaliado pelo ERF –, em 11 247 chineses, e identificaram razoável poder preditivo. As AUCs variaram entre 0,59 e 0,70 nas mulheres e entre 0,52 e 0,60 nos homens, resultados parecidos aos do presente estudo. Entretanto, os indicadores que apresentaram maiores AUC foram o *Body Shape Index* nos homens e a RCEst e o *Body Roundness Index* nas mulheres, indicadores não avaliados na presente análise.

Indicadores de distribuição da gordura corporal são apontados por alguns estudos^2^^,^^12^^,^^19^ como melhores preditores de risco à saúde do que indicadores de obesidade geral, pois são capazes de fornecer melhor estimativa da gordura abdominal, que, por sua vez, está correlacionada à quantidade de tecido adiposo visceral, e consequentemente, com a produção de maior quantidade de adipocinas pro-inflamatórias. Entretanto, o desempenho destes indicadores em estudos populacionais variou dependendo de características como idade, etnia e sexo, bem como, dos pontos de corte utilizados e do desfecho analisado.^19^^,^^23^^,^^34^ Situação similar ocorreu em estudos com os indicadores de obesidade geral,^10^^,^^14^^-^^16^ ou seja, componentes genéticos, ambientais e comportamentais de grupos étnicos distintos podem justificar as diferenças de desempenho encontradas, reforçando a necessidade de parâmetros mais apropriados e condizentes para triagem de risco à saúde de cada grupo populacional.

Outra questão a ser destacada é que não há consenso de que a combinação de indicadores antropométricos melhore o diagnóstico de desfechos adversos à saúde, e estudos que adotaram esta metodologia de análise são restritos.^19^^,^^23^^-^^26^ Especialistas continuam buscando melhores indicadores ou estratégias metodológicas que permitam rastreamentos mais precisos de agravos à saúde em diferentes populações. As combinações de, pelo menos, um indicador de obesidade geral e outro de obesidade central estiveram mais fortemente associadas a um maior risco de desenvolvimento de DAC, mesmo após ajuste pela presença de apenas um indicador geral ou central. Todas as combinações apresentaram maiores valores de sensibilidade e das AUC do que cada indicador isoladamente, sendo as combinações IAC+RCQ e IMC+RCQ, aquelas que apresentaram maiores AUC em homens e mulheres, respectivamente. Esses resultados sinalizam que essa metodologia de análise parece ser uma boa alternativa para melhorar o rastreamento de risco coronariano.

Mesmo considerando a possibilidade de certo grau de colinearidade entre os indicadores gerais e centrais, o que poderia indicar perda de magnitude do efeito, observou-se em todas as associações testadas que tanto RA10%, quanto RMA20% foram mais prevalentes entre os sujeitos que apresentaram valores elevados de um indicador geral com outro central (IAC_1_+CC_1_, IAC_1_+RCQ_1_, IMC_1_+CC_1_ e IMC_1_+RCQ_1_) do que a presença de apenas um indicador aumentado.

Os resultados de estudo conduzido por Lam et al.,^23^ corroboram em parte esses achados, ou seja, dois indicadores juntos aumentam a detecção de sujeitos verdadeiros-positivos com fatores de risco cardiometabólicos. No entanto, a combinação do IMC com a RCEst, tal como sugerido por estes autores, foi aquela com melhor desempenho em funcionários de um hospital em Singapura. Da mesma forma, Tao et al.,^25^ evidenciaram que dois indicadores juntos aumentaram a capacidade preditiva em até 19,45% do que apenas um indicador isoladamente. O IMC juntamente com a CC foi a combinação com maiores AUC para detectar hipertensão e síndrome metabólica em chineses de ambos os sexos, DM2 em homens e dislipidemia em mulheres. Da mesma forma, a união IMC+CC esteve mais fortemente relacionada com fatores de risco cardiovasculares em uma amostra de indivíduos de etnia branca do *Third National Health and Nutrition Examination Survey* (NHANES III), do que ambos indicadores isoladamente.^24^ Cabe ressaltar que esses dois últimos estudos não analisaram o IAC.

A principal força deste estudo consiste em ser o primeiro a testar combinações do IAC com outros indicadores de obesidade central para estimar risco coronariano, incluindo a análise de homens e mulheres de diferentes faixas etárias em separado. Os resultados apontaram que o uso combinado de um indicador de obesidade geral com outro de obesidade central propicia melhor identificação de pessoas ou populações adultas com maior risco de desenvolver risco coronariano em 10 anos. Além disso, o estudo apresenta comparações com testes estatísticos das AUCs individuais e combinadas dos indicadores entre os sexos e faixas etárias.

Entretanto, potenciais limitações devem ser discutidas. O desenho transversal não permite inferir sobre a relação causal entre medidas antropométricas de obesidade e risco coronariano futuro. Contudo, a causalidade reversa parece improvável e as evidências de que a obesidade é um importante fator do risco cardiovascular são consistentes.^5^^,^^20^^-^^22^ O acompanhamento dos participantes do ELSA-Brasil permitirá avaliar melhor se a combinação de indicadores de obesidade prediz melhor o risco coronariano do que o uso isolado de algum indicador. Outra limitação diz respeito à validade externa, pois, a amostra do ELSA-Brasil não é representativa da população brasileira, pois não inclui os segmentos extremos, como os mais ricos e mais pobres, por exemplo. No entanto, é uma amostra grande, multicêntrica e heterogênea o suficiente para trazer novos conhecimentos sobre a relação entre indicadores de obesidade geral e central com risco coronariano em populações miscigenadas. Portanto, novas investigações com outras populações, assim como pesquisas longitudinais e avaliação de outras combinações de indicadores são necessárias.

## Conclusão

A RCQ teve melhor desempenho individual em ambos os sexos e faixas etárias. As combinações, de pelo menos, um indicador de obesidade geral com outro de obesidade central, foram mais fortemente associadas a maior risco de desenvolvimento de DAC. As combinações IAC+RCQ e IMC+RCQ foram as que resultaram nas maiores AUCs para homens e mulheres, respectivamente. Assim, o uso combinado de um indicador de obesidade geral, IAC ou IMC, dependendo do sexo, com a RCQ é recomendado como estratégia para rastreamentos epidemiológicos de risco coronariano em adultos. Porém, em contextos de precariedade ou de indisponibilidade de uma balança para aferir o peso corporal, pode-se utilizar uma fita métrica para medir a CQ, a CC e a estatura, calcular o IAC e a RCQ e realizar a avaliação de risco, já que o desempenho desta combinação foi parecido com a combinação IMC+RCQ em mulheres.
