# Supplementary material for: Desempenho Individual e Combinado de Indicadores de Obesidade Geral e Central para Estimar Risco Coronariano em Participantes do ELSA-Brasil
Source: Arq Bras Cardiol. 2021 Jul 21;117(4):701–12. [Article in Portuguese] doi: 10.36660/abc.20200360 (PMC8528349; doi:10.36660/abc.20200360)
Supplement: Translation

# Translation: Individual and Combined Performance of Indicators of Overall and Central Obesity to Estimate Coronary Risk in ELSA-Brasil Participants

**Authors:** Rogério Tosta de Almeida, Sheila Maria Alvim Matos, Estela M. L. Aquino

**Affiliations:** 1 Feira de SantanaBA Brazil Universidade Estadual de Feira de Santana - Departamento de Saúde, Feira de Santana, BA – Brazil; 2 SalvadorBA Brazil Universidade Federal da Bahia - Instituto de Saúde Coletiva, Salvador, BA – Brazil

**Keywords:** Cardiovascular Disease, Anthropometry, Obesity, Risk Factors, Epidemiology, Adiposity, Body Mass Index, Waist Circumference

## Abstract

**Background:**

Anthropometric indicators have been used in clinical practice and epidemiological studies for screening of health risk factors.

**Objectives:**

To evaluate the individual discriminatory power of body adiposity index (BAI), body mass index (BMI), waist circumference (WC) and waist-hip-ratio (WHR) to identify individuals at risk for coronary heart disease and to evaluate whether combinations of anthropometric indicators of overall obesity with indicators of central obesity improve predictive ability in adults.

**Methods:**

A total of 15,092 participants (54.4% women) aged 35-74years were assessed at baseline of the ELSA-Brasil study. Individuals at risk for coronary heart disease were identified using the Framingham risk score and divided into very-high risk (VHR 20%) and high risk (HR10%). Measures of diagnostic accuracy and area under the ROC curves (AUC) were analyzed. Associations were tested using Poisson regression analysis with robust variance, according to age and sex. Statistical significance was set at 5%.

**Results:**

WHR showed the highest discriminatory power for VHR20% in all groups, with higher predictive ability in women (AUC: 0.802; 95%CI: 0.748-0.856 vs 0.657; 95%CI: 0.630-0.683 in the age range of 35-59 years, and AUC: 0.668; 95%CI: 0.621-0.715 vs 0.611; 95%CI: 0.587-0.635 in the age range of 60-74 years). BAI + WHR and BMI + WHR had the highest predictive power in men and women, respectively. Combinations of indicators of overall obesity with indicators of central obesity were more strongly associated with VHR20% and HR10% in all subgroups.

**Conclusion:**

Combined indicators had greater predictive ability than indicators taken individually. BAI+ WHR and BMI + WHR were the best estimators of coronary risk in men and women, respectively, and WHR had the best individual performance.

## Introduction

Anthropometric indicators of obesity have been widely used in clinical practice and epidemiological studies for the screening for health risk factors.^1^^-^^3^

Body mass index (BMI), devised by Lambert Adolphe Jaques Quetelet in 1832,^4^ is one of the most popular strategies used to indirectly measure obesity in populations. Although it has been extensively used in studies on cardiovascular diseases,^3^^,^^5^ this tool may not be able to describe the variation in body composition among individuals.^3^

In recent years, new indicators have been proposed as more accurate indirect measures of obesity. In 2011, Bergman et al.^6^ proposed the body adiposity index (BAI) as a better indicator of body fat as compared with BMI. However, the accuracy of this index in measuring adiposity has varied among populations,^7^^-^^9^ and results of its performance as a screening strategy for cardiometabolic risk factors are still controversial.^10^^-^^16^ Indicators of body fat distribution, waist circumference (WC), waist-to-hip ratio (WHR), waist-to-hip ratio (WHtR), and the conicity index (C index) have shown good performance as predictors of health risk.^12^^,^^17^ A recent study with participants of the ELSA-Brasil study demonstrated a positive association of WC and WHR with carotid intima-media thickness.^17^ In addition, the use of these indicators has been strengthened by epidemiological studies and does not require complex mathematical calculations.

It is controversial whether anthropometric indicators of overall and central obesity have higher predictive ability and usefulness to identify cardiometabolic risk factors in large populations.^18^^-^^22^ Besides, while some studies have shown that the combined use of indicators can improve their predictive capacity for adverse health outcomes,^18^^,^^23^^-^^25^ others have shown the opposite.^19^^,^^26^ Few studies evaluating the combined performance of indicators of overall and central obesity have been performed in the Brazilian adult population.

Therefore, the aims of the present study were to evaluate the individual discriminatory power of BAI, WC, WHR to assess coronary risk; to investigate whether the combination of anthropometric indicators of overall obesity (BMI and BAI) with indicators of central obesity (WC and WHC) improves the predictive ability of coronary risk in a large sample of Brazilian adults.

## Methods

### Source of data and study population

The Brazilian Longitudinal Study of Adult Health (ELSA-Brasil), evaluated, at baseline (2008-2010), 15,105 servants of educational and research institutions of six Brazilian cities (Belo Horizonte, Porto Alegre, Rio de Janeiro, Salvador, São Paulo and Vitória), aged between 35 and 74 years. Of these, nine did not have complete information on the variables analyzed, and four reported using silicone hip implants, and were then excluded. Thus, the present study investigated 15,092 patients. Methodological details of the ELSA-Brasil study were published by Aquino et al.^27^

### Demographic, anthropometric, and clinical data

Demographic, anthropometric, and clinical data were collected by interviews or measured, by trained personnel and supervised by highly qualified professionals. Age, sex, smoking and previous history of diabetes mellitus were collected using standardized procedures. Anthropometric measures were obtained after a 12-hour overnight fasting, with patients standing barefoot, wearing standard clothing provided by the ELSA-Brasil group. The measurements were taken based on a standardized protocol developed for the study, following the recommendations of the International Society for the Advancement of Kinanthropometry (ISAK).^28^

Weight was measured at the nearest 0.1 Kg using a calibrated scale (Toledo 2096PP). Height was measured using a stadiometer (Seca-SE0216), and WC and hip circumference measured at the nearest 0.1 cm using an inelastic tape. WC was measured at the midpoint between the lowest rib and the iliac crest, and HC was measured at the level of the greatest gluteus protuberance. Measures were taken in triplicate and the mean of the three measurements considered for analysis.

Blood samples were collected in dry tubes for determination of glycemia, and total cholesterol and fractions at the study centers after 12-hour overnight fasting. Plasma glucose levels were determined by a hexokinase (enzymatic) method, and cholesterol levels determined by the cholesterol oxidase method (enzymatic colorimetric method), both using standard calibrated equipment (Siemens ADVIA 1200). In order to ensure the quality and standardization of results, all samples were sent to ELSA-Brasil central laboratory for processing and analysis.

Three blood pressure measures were taken with a one-minute interval after five minutes of rest, using an automated device (OMRON – HEM-705 CP), with participants sitting in a quiet room at controlled temperature (20ºC - 24ºC), under fasting conditions and with an empty bladder, following the protocol developed for the study. The mean of the last two measurements was considered for analysis.

### Anthropometric indicators

The anthropometric indicators of obesity evaluated were BMI (Kg/m^2^), which was calculated by dividing weight (Kg) by height (m) squared, and BAI, calculated using the equation: BAI = ([hip circumference in cm / height in meters] 1.5)-18.^6^ Central obesity was estimated by measurements of WC (cm) and the WHR, the latter calculated by dividing WC (cm) by hip circumference (cm).

### Cardiovascular risk

The risk for cardiovascular events were calculated using the Framingham risk score (FRS), which included age, systolic blood pressure (SBP), systolic diastolic pressure (SDP), total cholesterol, high-density lipoprotein (HDL)-cholesterol, smoking and diabetes for its calculation.^29^

Diabetes was defined as a history of diabetes, fasting glucose ≥126 mg/dL, or a two-hour glucose ≥ 200 mg/dL (oral glucose tolerance test) or glycated hemoglobin ≥ 6.5%.

Current smokers were those who reported to smoke regularly in the interview.

An increased risk for cardiovascular events was classified as: “very high” (VH), for individuals with 20% or more 10-year coronary artery disease risk (VHR20%), of “high” for individuals with 10-20% 10-year coronary artery disease risk (HR10%).

### Statistical analysis

Central tendency and dispersion measures were used for initial analyses of patient distribution. Descriptive analysis was used to analyze normality of data, by graphic (histogram and P-Plot) and the Shapiro Wilk test. The F-test was used to analyze the homogeneity of variance. Continuous variables with normal distribution were expressed as mean and standard deviation and assessed using the unpaired t-Student test. Continuous variables with non-normal distribution were expressed as median and interquartile range and assessed by the Mann-Whitney U test. Categorical variables were expressed as absolute and relative frequencies and tested by the Pearson's chi-square test.

Then, the cutoff points for the anthropometric indicators (BMI, BAI, WHR and WC) to identify individuals at 20% or more 10-year coronary artery disease risk (VHR20%) were determined by ROC (Receiver Operating Characteristic) curves by age and sex. The maximum value of the Youden index (sensitivity + specificity – 1) was used as the criterion for selecting the optimum cut-off point. Areas under the ROC curves (AUCs) and respective 95% confidence intervals (95% CI) were used to evaluate individual predictive ability of the indicators. The AUCs were compared by the non-parametric test proposed by Delong et al.,^30^ and differences in the indicators alone and combined were compared between sexes and age ranges. Also, the AUC of the best combination of indicators was compared with that of the best individual indicator in each group.

Discriminatory ability of indicators was tested either alone or in combinations (indicator of overall obesity with indicator of central obesity. First, four combinations were analyzed (BAI + WC, BMI + WC, BAI + WHR and BMI + WHR), rated and grouped as follows: “0 = both indicators are within normal range”; “1 = indicator of overall obesity only is increased”; “2 = indicator of central obesity only is increased”; “3 = both indicators are increased”. For example, in the combination BAI + WC, the groups were: “0 = BAI_0_+WC_0_”; “1 = BAI_1_+WC_0_”; “2 = BAI_0_+WC_1_”; and “3 = BAI_1_+WC_1_”.

Subsequently, to determine the adjusted association of indicators of overall obesity with those of central obesity and combinations, another grouping into four categories was made, varying from none to at least one indicator (either overall or central obesity) was increased, i.e., 00, 01, 10, 11. For this purpose, dummies variables were created: 0 = participants without any increased indicator; 1 = participants with at least one indicator of overall obesity increased and indicators of central obesity within normal range; 2 = participants with at least one indicator of central obesity increased and indicators of central obesity within normal range; 3 = the other possible combinations between at least one indicator of central obesity increased and at least one indicator of overall obesity increased.

Associations were tested by Poisson regression models with robust variance, with analysis of the prevalence ratio (PR) and the 95% CI between the combinations of indicators and VHR20%. Due to the low frequency of VHR20% among young women (0.8%), and the small number of participants at risk in some combinations, an additional analysis was conducted to confirm or not the associations of the anthropometric indicators (alone or in combination) with cardiovascular risk, with HR10% as the outcome.

Model adjustments were assessed by analysis of Pearson residuals.

The significance level was set at 5% (p<0.05), and the statistical analyses were performed using the STATA software program version 12.0.

## Results

A total of 6,881 men (28.5% aged between 60 and 74 years) and 8,211 women (26.7% aged between 60 and 74 years) at baseline of the ELSA-Brasil study. Mean values of total cholesterol, SBP, DBP and median age and HDL-cholesterol were higher in participants of both sexes aged 60-74 years compared with younger ones, except DBP and total cholesterol in men.

The prevalence of VHR20% was 6.3% and 40.6% in men aged 35-59 years and 60-74 years, respectively, and 0.8% and 6.1% in women aged 35-59 years and 60-74 years, respectively. A higher prevalence of men with central obesity (WC_1_ and WHR_1_) was observed compared with women, in both age ranges, with higher prevalence in older (60-74 years) subjects of both sexes. Differences in the percentage of patients with overall obesity varied according to the indicator (Table 1).

**Table 1:** Characteristics of participants by sex and age range – ELSA-Brasil (2008-2010)

	Men		Women	
Variable	35-59 years (n= 5,354)	60-74 years (n= 1,527)	35-59 years (n= 6,478)	60-74 years (n= 1,733)
Age, median (IQR)	48 (44-54)*	65 (62-69)*†	49 (44-54)	64 (61-68)†
Total cholesterol, mean (SD)	214.1 (44.6)	207.1 (42.4)*†	214.5 (39.8)	224.2 (45.4)†
HDL-Cholesterol, median (IQR)	48 (42-56)*	50 (43-59)*†	59 (51-70)	62 (52-73)†
Systolic blood pressure, mean (SD)	124.0 (15.7)*	131.1 (19.2)*†	115.1 (15.3)	127.4 (19.1)†
Diastolic blood pressure, mean (SD)	79.2 (10.8)*	77.9 (10.9)*†	73.8 (10.2)	74.8 (10.2)†
HR,10%, n (%)	1.711 (32.0)*	1.285 (84.2)*†	542 (8.4)	613 (35.4%)†
VHR,20%, n (%)	340 (6.3)*	620 (40.6)*†	52 (0.8)	106 (6.1)†
Diabetes, n (%)	1.301 (19.3)*	569 (37.3)*†	882 (13.6)	484 (27.9)†
Smoking, n (%)	813 (15.2)*	171 (11.2)*†	855 (13.2)	138 (7.9)†
BAI_1,_ n (%)	1.521 (28.4)*	524 (34.3)†	2.468 (38.1)	585 (33.8)†
BMI_1_, n (%)	2.425 (45.3)*	542 (35.5)†	2.269 (35.0)	583 (33.6)
WC_1,_ n (%)	2.491 (46.5)*	871 (57.0)*†	2.346 (36.2)	682 (39.3)†
WHR_1,_ n (%)	1.761 (32.9)*	892 (58.4)*†	1.351 (20.9)	598 (34.5)†

HDL: high-density lipoprotein; HR10%: high risk (individuals with 10-20% 10-year coronary artery disease risk); VHR20%: very high risk (individuals with 20% or more 10-year coronary artery disease risk; BAI: body adiposity index; BMI: body mass index; WC: waist circumference; WHR: waist-hip ratio; SD: standard deviation; IQR: interquartile range; n (%): number of observations (frequency).

* p< 0.05 compared with women within the same age group;

† p< 0.05 compared with younger individuals of the same sex.

The best cutoff point of each indicator to detect VHR20% and define the groups is listed in Table 2, where 0 means “normal” and 1 “increased”.

**Table 2:** Cutoff points of anthropometric indicators and respective classifications – ELSA-Brasil (2008-2010)

Indicator	Cutoff point	Categories	Class*
BAI	28 (men) 34 (women 35-59 years) 36 (women 60-74 years)	BAI < 28 (men) or BAI < 34 (women 35-59 years) or BAI < 36 (women 60-74 years)	BAI_0_
		BAI ≥ 28 (men) or BAI ≥ 34 (women 35-59 years) or BAI ≥ 36 (women 60-74 years)	BAI_1_
BMI	27 (men 35-59 years) 28 (men 60-74 years) 28 (women 35-59 years) 29 (women 60-74 years)	BMI < 27 (men 35-59 years) or BMI < 28 (men 60-74 years) or BMI < 34 (women 35-59 years) or BMI < 36 (women 59-74 years)	BMI_0_
		BMI ≥ 27 (men 35-59 years) or BMI ≥ 28 (men 60-74 years) or BMI ≥ 34 (women 35-59 years) or BMI ≥ 36 (women 60-74 years)	BMI_1_
WC	95 (men) 90 (women 35-59 years) 93 (women 60-74 years)	WC < 95 (men) or WC < 90 (women 35-59 years) or WC < 93 (women 60-74 years)	WC_0_
		WC ≥ 95 (men) or WC ≥ 90 (women 35-59 years) or WC ≥ 93 (women 60-74 years)	WC_1_
WHR	0.97 (men) 0.90 (women 35-59 years) 0.91 (women 60-74 years)	WC < 0.97 (men) or WC < 0.90 (women 35-59 years) or WC < 0.91 (women 60-74 years)	WHR_0_
		WC ≥ 0.97 (men) or WC ≥ 0.90 (women 35-59 years) or WC ≥ 0.91 (women 60-74 years)	WHR_1_

BAI: body adiposity index; BMI: body mass index; WC: waist circumference; WHR: waist-hip ratio;

* “0 = normal” and “1 = increased”.

Among the anthropometric indicators, WHR showed the highest discriminatory ability for VHR20% in all groups. The AUC for the combination BAI + WHR was higher in men and the AUC for BMI + WHR was higher in women in both age ranges. The performance of all indicators, either alone or in combinations, was better in women than men, except the BAI_1_ that did not show statistically significant difference. Likewise, in the comparison of participants between age groups, only BAI_1_ in both sexes and BMI_1_ in men did not show statistically significant difference.

Compared with individual analyses of the indicators, combined analyses yielded higher sensitivity, except for the BAI + WC and BMI + WC combinations in older men, which had lower sensitivity than the WHR. Sensitivity of all combinations were higher than 70%, reaching values higher than 90% (Table 3) in women aged 35-59 years (Table 3).

**Table 3:** Measures of diagnostic accuracy of individual and combined anthropometric indicators of obesity to estimate 20% of coronary risk in 10 years, according to sex and age group. ELSA-Brasil (2008-2010).

	Men 35-59 years (n=5,354)			Men 60-74 years (n=1,527)			
Indicators	SEN (%)	SPE (%)	AUC (95%CI)	SEN (%)	SPE (%)	AUC (95%CI)	p
**Individual**							
*BAI_1_*	43.2	72.6	0.578 (0.551 – 0.605)	39.8	69.5	0.547 (0.522 – 0.571)	0.080
*BMI_1_*	62.9	55.9	0.594 (0.568 – 0.621)	43.2	69.8	0.565 (0.541 – 0.590)	0.116
*WC_1_*	64.9	54.7	0.598 (0.572 – 0.624)	64.2	47.9	0.560 (0.535 – 0.585)	0.041
*WHR_1_*	62.2	69.1	**0.657 (0.630 – 0.683)** a b c	71.6	50.6	**0.611 (0.587 – 0.635)** a b c	0.013
Combinations							
*BAI + WC*	70.9	49.3	0.618 (0.589 – 0.647)	70.3	41.7	0.574 (0.549 – 0.601)	0.029
*BAI + WHR*	75.3	55.7	**0.680 (0.652 – 0.707)**^1^.^3^#	80.3	40.0	**0.624 (0.597 – 0.650)**^1^.^3^	0.004
*BMI + WC*	71.2	48.3	0.610 (0.583 – 0.638)	64.6	46.6	0.578 (0.550 – 0.605)	0.010
*BMI + WHR*	77.9	48.4	0.672 (0.645 – 0.700)^2^.^4^	76.0	44.4	0.623 (0.596 – 0.650)^2^.^4^	0.013
	**Women 35-59 years (n=6,478)**			**Women 60-74 years (n=1,733)**			
**Indicators**	**SEN (%)**	**SPE (%)**	**AUC (95%CI)**	**SEN (%)**	**SPE (%)**	**AUC (95%CI)**	**p**
**Individual**							
*BAI_1_*	63.5	62.1	0.628 (0.562 – 0.694)	50.9	67.4	0.592 (0.542 – 0.641)	0.389
*BMI_1_*	78.9	65.3	0.721 (0.665 – 0.777)^e^*	58.5	68.0	0.632 (0.584 – 0.681)*	0.020
*WC_1_*	92.3	64.2	0.783 (0.746 – 0.820)^d^^f^*	68.9	62.6	0.657 (0.611 – 0.703)^d^*	<0.001
*WHR_1_*	80.8	79.6	**0.802 (0.748 – 0.856)** a b *	66.0	67.6	**0.668 (0.621 – 0.715)** a *	<0.001
Combinations							
*BAI + WC*	94.2	51.6	0.777 (0.734 – 0.820)*	75.5	52.6	0.667 (0.618 – 0.717)*	0.001
*BAI + WHR*	98.1	53.2	0.846 (0.810 – 0.882)^1^.^3^*	85.8	48.1	0.709 (0.663 – 0.754)*	<0.001
*BMI + WC*	92.3	59.0	0.786 (0.743 – 0.829)*	70.8	57.9	0.668 (0.618 – 0.717)*	<0.001
*BMI + WHR*	96.2	59.4	**0.850 (0.811 – 0.889)**^2^.^4^*#	82.1	52.1	**0.710 (0.663 – 0.757)** 4 * #	<0.001

BAI: body adiposity index; BMI: body mass index; WC: waist circumference; WHR: waist-hip ratio; 95%CI: 95% confidence interval; p: p value when comparing age groups of the same sex. Bold: Highest AUC values in each grouping. p≤0.05 between individual indicators by sex – SEN: sensitivity; SPE: specificity; AUC: Area under the ROC curves.

a WHR > BAI;

b WHR > BMI;

c WHR > WC;

d WC > BAI; and BMI> BAI;

f WC > BMI; between combinations by sex

1 BAI+WHR > BAI+WC;

2 BMI+WHR > BAI+WC;

3 BAI+WHR > BMI+WC;

4 BMI+WHR > BMI+WC.

* with men of the same age group;

# best match with best individual.

Combinations of two indicators had greater predictive capacity than the indicators alone. For example, while AUCs of BMI_1_ and WHR_1_ in women aged 60-74 years were 0.632 and 0.668, respectively, the AUC of BMI + WHR was 0.710, i.e., an increase of at least 0.042 (0.042/0.668 = 6.3%). The increment with the best combinations varied from 2.1% to 6.3% (Figure 1).

**Figure 1:** Graphic representation of areas under the ROC curve of individual and combined anthropometric indicators to identify very-high risk for coronary artery disease by sex and age ranges, ELSA-Brasil 2008-2010.

Combinations of one indicator of overall obesity with one indicator of central obesity (BAI_1_+WC_1_, BAI_1_ + WHR_1_, BMI_1_+WC_1_ and BMI_1_+WHC_1_) were positively associated with VHR20% in men and women in both, regardless of the presence of only one indicator, be it of overall or central obesity. The prevalence ratios of these combinations were also higher than in all other groups (Table 4).

**Table 4:** Adjusted prevalence ratios of combinations of anthropometric indicators of obesity to estimate very-high risk for coronary artery disease, by sex and age ranges – ELSA-Brasil (2008-2010)

	Men				Women			
	35-59 years (n=5,354)		60-74 years (n=1,527)		35-59 years (n=6,478)		60-74 years (n=1,733)	
Combinations of indicators	n (risk)	PR (95%CI)	n (risk)	PR (95%CI)	n (risk)	PR (95%CI)	n (risk)	PR (95%CI)
BAI_0_ + WC_0_	2,573 (99)	1.0	562 (184)	1.0	3,321 (3)	1.0	881 (26)	1.0
BAI_1_ + WC_0_	290 (20)	1.79 (1.13 – 2.85)	94 (38)	1.23 (0.94 – 1.62)	811 (1)	1.36 (0.14 – 13.11)	170 (7)	1.40 (0.62 – 3.16)
BAI_0_ + WC_1_	1,260 (94)	1.94 (1.47 – 2.55)	441 (189)	1.31 (1.12 – 1.54)	689 (16)	25.70 (7.51 – 87.99)	267 (26)	3.30 (1.95 – 5.58)
BAI_1_ + WC_1_	1,231 (126)	2.66 (2.06 – 3.43)	430 (209)	1.48 (1.27 – 1.73)	1,657 (32)	21.38 (6.55 – 69.72)	415 (47)	3.84 (2.41 – 6.11)
								
BAI_0_ + WHR_0_	2,876 (84)	1.0	485 (122)	1.0	3,420 (1)	1.0	798 (15)	1.0
BAI_1_ + WHR_0_	717 (44)	2.10 (1.47 – 3.00)	150 (54)	1.43 (1.10 – 1.86)	1,707 (9)	18.03 (2.29 – 142.22)	337 (21)	3.32 (1.73 – 6.35)
BAI_0_ + WHR_1_	957 (109)	3.90 (2.96 – 5.13)	518 (251)	1.93 (1.61 – 2.30)	590 (18)	104.33 (13.95 – 780.18)	350 (37)	5.62 (3.13 – 10.11)
BAI_1_ + WHR_1_	804 (102)	4.34 (3.29 – 5.74)	374 (193)	2.05 (1.71 – 2.46)	761 (24)	107.85 (14.62 – 796.13)	248 (33)	7.08 (3.91 – 12.82)
								
BMI_0_ + WC_0_	2,521(98)	1.0	642 (219)	1.0	3,795 (4)	1.0	973 (31)	1.0
BMI_1_ + WC_0_	342 (21)	1.58 (1.00 – 2.50)	14 (3)	0.63 (0.23 – 1.72)	337 (0)	----	78 (2)	0.80 (0.20 – 3.30)
BMI_0_ + WC_1_	408 (28)	1.76 (1.17 – 2.65)	343 (133)	1.14 (0.96 – 1.35)	414 (7)	16.04 (4.71 – 54.61)	177 (13)	2.31 (1.23 – 4.32)
BMI_1_ + WC_1_	2,083 (192)	2.37 (1.87 – 3.00)	528 (265)	1.47 (1.28 – 1.69)	1,932 (41)	20.14 (7.22 – 56.19)	505 (60)	3.73 (2.45 - 5.68)
								
BMI_0_ + WHR_0_	2,500 (75)	1.0	552 (149)	1.0	3,820 (2)	1.0	866 (19)	1.0
BMI_1_ + WHR_0_	1,093 (53)	1.62 (1.15 – 2.28)	83 (27)	1.21 (0.86 – 1.69)	1,307 (8)	11.69 (2.48 – 54.99)	269 (17)	2.88 (1.52 – 5.46)
BMI_0_ + WHR_1_	429 (51)	3.96 (2.82 – 5.57)	433 (203)	1.74 (1.46 – 2.06)	389 (9)	44.19 (9.58 – 203.82)	284 (25)	4.01 (2.24 – 7.18)
BMI_1_ + WHR_1_	1,332 (160)	4.00 (3.07 – 5.22)	459 (241)	1.95 (1.65 – 2.19)	962 (33)	65.52 (15.75 – 272.60)	314 (45)	6.53 (3.88 – 10.99)

BAI: body adiposity index; BMI: body mass index; WC: waist circumference; WHR: waist-hip ratio; PR: prevalence ratio; 95%CI: 95% confidence interval; n: number of participants in the subgroup; risk: number of participants at very-high risk for coronary events

Although the prevalence of women with VHR20% was low (<1%) in the group aged 35-59 years, the number of women taken as reference (without risk) was very small, which may have explained the wide confidence intervals. Besides, in some strata, the number of participants at risk was small, as in the combination BMI_1_+WC_0_ in elderly men and women, which made difficult the analyses in these groups. For this reason, we also evaluated the associations of the combinations of anthropometric indicators with HR10%, which confirmed the results that individuals with an increased indicator of overall obesity (BAI or BMI) and an increased indicator of central obesity (WC or WHR) have higher 10-year coronary disease risk (Table 5).

**Table 5:** Adjusted prevalence ratios of combinations of anthropometric indicators of obesity to estimate high risk for coronary artery disease, by sex and age ranges – ELSA-Brasil (2008-2010)

	Men				Women			
	35-59 years (n=5,354)		60-74 years (n=1,527)		35-59 years (n=6,478)		60-74 years (n=1,733)	
Combinations of indicators	n (risk)	PR (95%CI)	n (risk)	PR (95%CI)	n (risk)	PR (95%CI)	n (risk)	PR (95%CI)
**BAI_0_ + WC** **_0_**	2,573 (559)	1.0	562 (430)	1.0	3,321 (137)	1.0	881 (213)	1.0
**BAI_1_ + WC** **_0_**	290 (94)	1.49 (1.24 – 1.79)	94 (77)	1.07 (0.96 – 1.19)	811 (35)	1.05 (0.73 – 1.50)	170 (49)	1.19 (0.92 – 1.55)
**BAI_0_ + WC** **_1_**	1,260 (485)	1.77 (1.60 – 1.96)	441 (386)	1.14 (1.08 – 1.21)	689 (106)	3.73 (2.93 – 4.74)	267 (132)	2.04 (1.73 – 2.42)
**BAI_1_ + WC** **_1_**	1,231 (572)	2.14 (1.94 – 2.35)	430 (209)	1.19 (1.13 – 1.26)	1,657 (264)	3.86 (3.17 – 4.71)	415 (219)	2.18 (1.88 – 2.53)
								
**BAI_0_ + WHR** **_0_**	2,876 (607)	1.0	485 (345)	1.0	3,420 (125)	1.0	798 (161)	1.0
**BAI_1_ + WHR** **_0_**	717(239)	1.58 (1.39 – 1.79)	150 (127)	1.19 (1.09 – 1.30)	1,707 (117)	1.88 (1.47 – 2.40)	337 (122)	1.79 (1.47 – 2.19)
**BAI_0_ + WHR** **_1_**	957 (437)	2.16 (1.96 – 2.39)	518 (471)	1.28 (1.20 – 1.36)	590 (118)	5.47 (4.32 – 6.93)	350 (184)	2.61 (2.20 – 3.09)
**BAI_1_ + WHR** **_1_**	804 (427)	2.52 (2.29 – 2.77)	374 (342)	1.29 (1.20 – 1.37)	761 (182)	6.54 (5.28 – 8.10)	248 (146)	2.92 (2.45 – 3.47)
								
**BMI_0_ + WC** **_0_**	2,521 (562)	1.0	642 (498)	1.0	3,795 (146)	1.0	973 (233)	1.0
**BMI_1_ + WC** **_0_**	342 (91)	1.19 (0.99 – 1.44)	14 (9)	0.83 (0.56 – 1.23)	337 (26)	2.00 (1.34 – 3.00)	78 (29)	1.55 (1.14 – 2.12)
**BMI_0_ + WC** **_1_**	408 (154)	1.69 (1.47 – 1.96)	343 (299)	1.12 (1.06 – 1.19)	414 (57)	3.58 (2.68 – 4.78)	177 (85)	2.01 (1.66 – 2.42)
**BMI_1_ + WC** **_1_**	2,083 (903)	1.94 (1.78 – 2.12)	528 (479)	1.17 (1.11 – 1.23)	1,932 (313)	4.21 (3.49 – 5.09)	505 (266)	2.20 (1.91 – 2.53)
								
**BMI_0_ + WHR** **_0_**	2,500 (512)	1.0	552 (401)	1.0	3,820 (131)	1.0	866 (172)	1.0
**BMI_1_ + WHR** **_0_**	1,093 (334)	1.49 (1.33 – 1.68)	83 (71)	1.18 (1.06 – 1.30)	1,307 (111)	2.48 (1.94 – 3.16)	269 (111)	2.08 (1.71 – 2.53)
**BMI_0_ + WHR** **_1_**	429 (204)	2.32 (2.05 – 2.63)	433 (396)	1.26 (1.19 – 1.34)	389 (72)	5.40 (4.13 – 7.06)	284 (146)	2.59 (2.17 – 3.08)
**BMI_1_ + WHR** **_1_**	1,332 (660)	2.42 (2.20 – 2.66)	459 (417)	1.25 (1.18 – 1.33)	962 (228)	6.91 (5.64 – 8.47)	314 (184)	2.95 (2.51 – 3.47)

BAI: body adiposity index; BMI: body mass index; WC: waist circumference; WHR: waist-hip ratio; PR: prevalence ratio; 95%CI: 95% confidence interval; n: number of participants in the subgroup; risk: number of participants at very-high risk for coronary events

The presence of central obesity (WC_1_ and/or WHR_1_), in absence of overall obesity (BMI_1_ and/or BAI_1_) was more strongly associated with VHR20% and HR10% than the reverse combination. The magnitude of the effect of the combined presence of overall and central obesity was greater in all strata, except in the association with VHR20% in younger men (Table 6).

**Table 6:** Prevalence ratios of anthropometric indicators of overall and central obesity to estimate very high and high 10-year coronary artery disease risk by sex and age range – ELSA-Brasil (2008-2010)

Very-high risk (VHR20%)								
	Men				Women			
	35-59 years (n=5,354)		60-74 years (n=1,527)		35-59 years (n=6,478)		60-74 years (n=1,733)	
Combinations of indicators	n (risk)	PR (95%CI)	n (risk)	PR (95%CI)	n (risk)	PR (95%CI)	n (risk)	PR (95%CI)
Without risk	2,201 (63)	1.0	421 (111)	1.0	3,079 (1)	1.0	721 (13)	1.0
Overall obesity only	438 (23)	1.83 (1.15 – 2.92)	81 (28)	1.31 (0.93 – 1.84)	850 (1)	3.62 (0.23 – 57.79)	168 (7)	2.31 (0.97 – 5.70)
Central obesity only	585 (55)	3.28 (2.31 – 4.66)	440 (199)	1.72 (1.42 – 2.07)	509 (8)	48.39 (6.06 – 386.16)	303 (25)	4.58 (2.37 – 8.83)
Central + Overall	2,130 (198)	3.25 (2.46 – 4.28)	585 (282)	1.83 (1.53 – 2.19)	2,039 (42)	63.42 (8.73 – 460.51)	541 (61)	6.25 (3.47 – 11.26)
								
**High risk (HR10%)**								
Without risk	2,201 (425)	1.0	421 (300)	1.0	3,079 (103)	1.0	721 (135)	1.0
Overall obesity only	438 (118)	1.40 (1.17 – 1.66)	81 (63)	1.09 (0.96 – 1.24)	851 (30)	1.05 (0.71 – 1.57)	168 (48)	1.53 (1.15 – 2.03)
Central obesity only	585 (242)	2.14 (1.88 – 2.47)	440 (393)	1.25 (1.17 – 1.34)	509 (80)	4.70 (3.56 – 6.20)	303 (144)	2.54 (2.09 – 3.08)
Central + Overall	2,130 (925)	2.25 (2.04 – 2.48)	585 (529)	1.27 (1.19 – 1.36)	2,039 (329)	4.82 (3.89 – 5.98)	541 (286)	2.82 (2.38 – 3.35)

PR: prevalence ratio; 95%CI: 95% confidence interval; n: number of participants in the subgroup; risk: number of participants at risk for future coronary events

## Discussion

BAI_1_ showed a modest predictive ability in all groups, with an AUC varying between 0.547 (95%CI: 0.522-0.571) in men aged 60-74 years and 0.628 (95%CI:0.562-0.694) in younger women. This result was similar to BMI_1_, since despite the slightly greater AUC values compared with the AUCs of BAI_1_ only in the group of younger women, such difference was statistically significant (p<0.05). Of all anthropometric indicators analyzed, the WHR was the one with the best individual performance.

The ROC curves are frequently used to evaluate the performance of a diagnostic test, and probably the most used statistical analysis to measure the predictive ability of anthropometric indicators. Many studies, however, instead of performing a statistical comparison between indicators or the groups, they have been restricted to observing the largest AUC, making it difficult to evaluate its performance. In a Chinese study by Xiao et al.,^12^ the AUCs were compared, showing that the discriminatory abilities of type 2 diabetes mellitus (DM2), BAI and BMI were not statistically different between men and women. Also, central obesity indicators – WC, WHtR and WHR – were better predictors than DM2 in women than in men.^12^ These findings are similar to our results that demonstrated a comparable performance of BMI and BAI in both sexes, and that both WC and WHR were better predictors of coronary risk in women.

Leal Neto et al.^14^ did not find statistically significant differences between the AUCs of BAI and BMI (p=0.885) in discriminating hypertension in Brazilian elderly (men and women), despite slightly larger AUCs of BAI than BMI. Similar results in the comparison of the discriminatory ability of BAI and BMI were reported in a Colombian study,^10^ in identifying elevated blood pressure and glycemia, and metabolic syndrome in women,^10^ and in identifying hypertension in a sample of adult men participating in the Olivetti Heart Study.^31^ Nevertheless, BMI was a better discriminator of metabolic syndrome in Korean women.^32^

On the other hand, Alvim et al.^13^ pointed out that BAI, compared with WC and BMI, was a better predictor of DM2 in male and female Amerindians in Brazil. However, in a sample of the general population, residents of a capital city, the performance of BAI, BMI and WC was similar in men and WC was a better predictor of DM2 than BAI and BMI in women.

A study conducted with 2,981 Iranians, BAI and BMI had comparable performance as predictors of DM2 in men, whereas BAI was not a good predictor in women (AUC=0.527; 95%CI: 0.484-0.569). A reasonable predictive power of WHR was seen in both men and women; worse than WC and WHtR in men and similar to the other indicators in women.^33^

Few studies exist investigating global coronary risk as an outcome, which makes comparison of our results with others difficult. However, Felix-Redondo et al.,^22^ in a study with 28,743 individuals from Spain, reported a positive association of BMI, WC and WHR with future coronary events, calculated by the REGICOR score, adapted from the FRS and validated for the Spanish population. Almeida et al.^15^ identified a positive association between BAI and FRS in 14,673 participants of the ELSA-Brasil study.

Wang et al.,^34^ evaluated the individual performance of eight anthropometric indicators to estimate coronary risk (using the FRS) in 11,247 Chinese people, and identified a reasonable predictive power. The AUCs varied between 0.59 and 0.70 in women and between 0.52 and 0.60 in men, which was similar to our results. However, largest AUCs were found for the Body Shape Index in men and the WHtR and Body Roundness Index in women, which was not evaluated in the present study.

Some studies^2^^,^^12^^,^^19^ have suggested that indicators of body fat distribution are better predictors of health risk than indicators of overall obesity, as they provide a better estimate of abdominal fat, which is correlated with the amount of visceral adipose tissue, and consequently to greater production of pro-inflammatory adipokines. Nevertheless, the performance of these indicators in population studies has varied depending on characteristics like age, ethnicity, and sex, as well as on the cut-off points and outcomes analyzed.^19^^,^^23^^,^^34^ Likewise, studies^10^^,^^14^^-^^16^ on indicators of overall obesity have shown that genetic, environmental and behavioral components of different ethnic groups may explain differences in their performance, reinforcing the need for more appropriate parameters for screening health risk factors in each population.

In addition, there is no consensus that the combination of anthropometric indicators improves the diagnosis of adverse health effects, and studies using this methodology are scarce.^19^^,^^23^^-^^26^ Experts continue to search for better indicators or methodological strategies aiming at more accurate screening for diseases in different populations. Combinations of at least one indicator of overall obesity with one indicator of central obesity were more strongly associated with an increased risk of coronary artery disease, even after adjustments for the presence of either one indicator of overall obesity or of central obesity. All combinations showed higher sensitivity and larger AUCs than the indicators analyzed individually. The combinations BAI + WHR and BMI + WHR showed the largest AUC for men and women, respectively. These findings indicate that this method of analysis seems to be a good alternative to improve the screening for coronary risk factors.

Even considering certain degree of collinearity between indicators of overall and central obesity, which would indicate a reduction in the magnitude of the effect, in all associations, both HR10% and VHR20% were more prevalent in those individuals with increased values of one indicator of overall obesity and one indicator of central obesity concomitantly (BAI_1_+WC_1_, BAI_1_+WHR_1_, BMI_1_+WC_1_, and BMI_1_+WHR_1_) than the presence of only one indicator increased.

The study by Lam et al.^23^ partly corroborate these results in that two indicators combined improve the detection of individuals with cardiometabolic risk factors. However, in their study, the combination of BMI with WHtC, was the one with the best performance in employees of a hospital in Singapore. Likewise, Tao et al.^25^ showed that two indicators combined improved the predictive ability by up to 19.45% as compared with one indicator alone. The combination of BMI plus WC was the one with the largest AUC to detect hypertension and metabolic syndrome in Chinese men and women, DM2 in men and dyslipidemia in women. Also, this combination was more strongly associated with cardiovascular risk factors in a sample of white individuals from the Third National Health and Nutrition Examination Survey (NHANES III) than any of the indicators alone.^24^ It is worth mentioning that these two last studies did not evaluate BAI.

The main strength of the present study is the fact that this is the first study to test combinations of BAI with other indicators of central obesity to estimate coronary risk, including separate analysis of men and women in different age ranges. The results indicated that the combined use of an indicator of overall obesity with another of central obesity improves the identification of adult people or populations at higher 10-year risk of coronary events. Also, the study presents statistical comparisons of AUCs of the indicators (individually and combined) between sexes and age ranges.

However, there are potential limitations that should be considered. First, the cross-sectional design of the study does not allow inferences about the causal relationship between anthropometric measures of obesity and future coronary risk. Nevertheless, a reverse causality seems improbable, and strong evidence supports that obesity is a risk factor for cardiovascular disease.^5^^,^^20^^-^^22^ The follow-up of participants of the ELSA-Brasil study will allow to assess whether the combination of obesity indicators is a better predictor of coronary risk than the indicator alone. Another limitation is the external validity, since the sample population from the ELSA-Brasil study is not representative of the Brazilian population, as it does not include extreme segments such as the richer and poorer income groups. However, this is a large, multicenter and heterogenous sample, able to provide new insights of the relationship of overall and central obesity indicators with coronary risk in heterogeneous populations. Therefore, further investigations with other populations, as well as longitudinal studies and examination of other combinations of obesity indicators are warranted.

## Conclusion

The WHR showed the best individual performance in both sexes and both age ranges. Combinations of at least one indicator of overall obesity with another of central obesity were more strongly associated with the risk of coronary artery disease. The combinations BAI+WHR and BMI + WHR yielded the largest AUCs in men and women, respectively. Thus, the combined use of one indicator of overall obesity (BAI or BMI, according to sex) with WHR is recommended as a screening strategy for coronary risk in adults. However, in low-income settings or in cases where a weighing scale for body weight is not available, a measuring tape can be used to measure hip circumference, WC and height, calculate BAI and WHR and perform risk assessment, since the performance of this combination was comparable to that of BMI + WHR in women.
